# Derivation of Water Quality Criteria for Carbamazepine and Ecological Risk Assessment in the Nansi Lake Basin

**DOI:** 10.3390/ijerph191710875

**Published:** 2022-08-31

**Authors:** Jiangyue Wu, Dianlong Shi, Sai Wang, Xi Yang, Hui Zhang, Ting Zhang, Lei Zheng, Yizhang Zhang

**Affiliations:** 1 National Marine Hazard Mitigation Service, Ministry of Natural Resource of the People’s Republic of China, Beijing 100194, China; 2 State Environmental Protection Key Laboratory of Dioxin Pollution, National Research Center of Environmental Analysis and Measurement, Sino-Japan Friendship Centre for Environmental Protection, Beijing 100029, China; 3 Chinese Research Academy of Environmental Sciences, Beijing 100012, China; 4 Research Institute for Environmental Innovation (Tianjin Binhai), Tianjin 300457, China

**Keywords:** carbamazepine, Nansi Lake basin, spatial distribution, species sensitivity distributions, water quality criteria, risk assessment

## Abstract

Carbamazepine, as one of several pharmaceutical and personal care products, has gained much attention in recent years because of its continuous discharge in natural waters and toxicity to aquatic ecosystems. However, it is difficult to evaluate and manage carbamazepine pollution because of the lack of a rational and scientific Water Quality Criteria (WQC) of carbamazepine. In this study, the carbamazepine toxicity data of thirty-five aquatic species from eight taxonomic groups were selected, and the species sensitivity distribution (SSD) method was applied to derive the WQC for carbamazepine based on the Log-logistic model, which was 18.4 ng/L. Meanwhile, the occurrence and distribution of carbamazepine in the Nansi Lake basin was studied. Results showed that concentrations of carbamazepine in 29 sampling sites were in the range of 3.3 to 128.2 ng/L, with the mean of 17.3 ng/L. In general, the levels of carbamazepine in tributaries were higher than those in the lakes. In addition, qualitative and quantitative ecological risk assessment methods were applied to assess the adverse effect of carbamazepine on aquatic systems. The hazard quotient (HQ) method showed that there were 24 and 5 sampling sites, in which risk levels were low and moderate, respectively. The joint probability curve (JPC) method indicated that ecological risks might exist in 1.4% and 1.0% of surface water, while a 5% threshold and 1% threshold were set up to protect aquatic species, respectively. Generally, carbamazepine posed a low risk to the aquatic organisms in the Nansi Lake basin.

## 1. Introduction

Pharmaceuticals and personal care products (PPCPs), as one of the most important groups of emerging contaminants, have become a global research hotspot due to their potential threats to the aquatic ecosystem and human health in recent years [1,2]. Currently, because of its daily or even excessive consumption for treating epilepsy and nerve pain, long half-life (900 h), and the low processing efficiency in the sewage treatment plant, carbamazepine is one of the most frequently detected PPCPs in the aquatic environment, and has been successfully proved to be a typical indicator that is able to trace the source of PPCPs in a large number of countries and regions [3,4,5]. Meanwhile, several researchers have introduced the fact that the overuse of carbamazepine can significantly affect aquatic animals both biochemically and physiologically [6,7,8]. Jarvis et al. [9] also reported that long-time exposure to carbamazepine could affect the abundance of Cyclopoida via the aquatic environment. Consequently, carbamazepine has been added to the priority list of the European Demonstration Program due to their frequency and toxicity [10]. However, carbamazepine, as an emerging pollutant, has not yet been included in the environmental quality standard system for surface water by America, China, and other countries. Therefore, it is very necessary and urgent to derive the Water Quality Criteria (WQC) of carbamazepine and to assess its ecological risk to aquatic system.

Water Quality Criteria (WQC) provides fundamental information of surface water for setting an environmental quality standard, ecological risk assessment, and environmental management [11]. Therefore, WQC research has been regarded as an important indicator, reflecting the progress of the environmental science research of one country. WQC-related studies have been carried out for several decades in developed countries and regions, such as the United States, the European Union, and Australia. In these areas, existing WQC guidance documents and related methodological systems are relative systematic and comprehensive [12,13,14,15,16,17]. However, WQC studies have just emerged in China, and the systematic research has only began in recent years. Based on the international derivation method of WQC, Chinese scholars have investigated and derived the WQC of target pollutants in Chinese surface water, such as ammonia-nitrogen [18], trivalent and pentavalent arsenic [19], nitrobenzene [20], plasticizer [21], and various heavy metals [22]. At the national level, the Ministry of Ecology and Environment issued a technical guideline for deriving the water quality criteria for the protection of freshwater aquatic organisms in 2017 by consulting the methodology of WQC derivation from developed countries [23] and revised the technical guideline in 2022 [24]. The undergoing WQC research in China is on the right track.

In this study, concentration and distribution of carbamazepine were analyzed in the Nansi Lake basin. By collecting and screening the toxicity data of carbamazepine, the WQC of carbamazepine was derived using the species sensitivity distribution (SSD) method. In addition, a tiered ecological risk assessment for carbamazepine was carried out by using qualitative and quantitative analysis. Taking carbamazepine as an example, the environmental investigation and evaluation system of merging pollutants was established preliminarily through field sampling, laboratory analysis, WQC derivation, and risk assessment.

## 2. Materials and Methods

### 2.1. Environmental Concentrations

#### 2.1.1. Study Area

The Naisi Lake (34°27′–35°20′ N, 116°34′–117°21′ E) is the largest shallow freshwater lake in China, which is also a real-time storage reservoir on the East Route of the South-to-North Water Diversion Project. It consists of four small lakes, namely, the Weishan Lake, Zhaoyang Lake, Dushan, Lake and Nanyang Lake, from south to north. The total drainage area of the Nansi Lake basin is 30,453 km^2^, and the water area is 1266 km^2^, with an average depth of 1.46 m [25]. The Nansi Lake Basin has a warm, temperate continental monsoon climate, with an average annual precipitation of 690 mm. Moreover, it is also one of the most important agricultural regions in the Shandong province of China, with major cash crops such as corn, rice, wheat, soybean, and cotton [26].

#### 2.1.2. Sample Collection and Extraction

**Figure 1:**
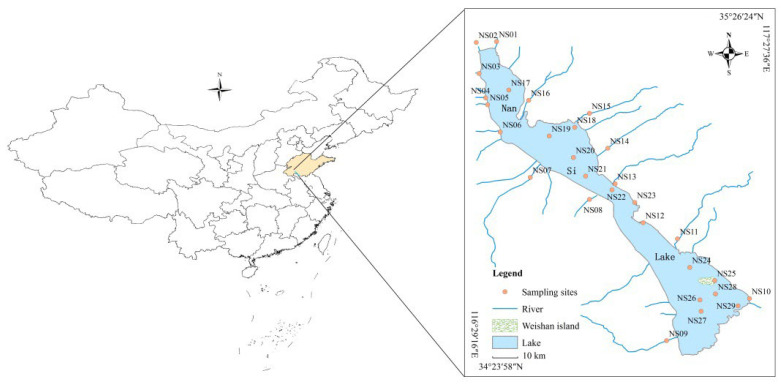
Sampling sites in the Nansi Lake basin.

A total of 29 surface water samples (NS01-SN29) were collected from the Nansi Lake basin in 2019 (Figure 1). The samples were taken from 0.5 m below the water surface, given a recovery indicator of carbamazepine-D8 (50 ng), and then filtered by a 70 mm GF/F glass fiber membrane within 24 h [27]. A solid-phase system (SPE) was used to extract carbamazepine with Oasis HLB cartridges which were pre-activated with 5 mL methanol and 5 mL ultra-pure water. The Oasis HLB cartridges absorbing carbamazepine were dried by vacuum (1.0 h) and eluted by 12 mL methanol. The eluent was concentrated 1.0 mL by high pure nitrogen and then spiked with caffeine-D3 as internal standard. Finally, the levels of carbamazepine in all samples were detected by using a high-performance liquid chromatography–triple quadrupole tandem mass spectrometry (HPLC-MS/MS, Waters, USA, Table S1) [28,29].

#### 2.1.3. Quality Assurance and Quality Control

All chemical analyses were strictly conducted following the quality assurance and quality control procedures. In this study, six different carbamazepine standard solutions (1.0–1000 ng/L) were applied to construct a calibration curve with a good linearity (*r*^2^ > 0.999). A solvent blank, a procedure blank, and an intermediate concentration of calibration curve were conducted for every bench of ten samples in order to check interference and cross-contamination. The method detection limit (MDL) of carbamazepine was 1.0 ng/L, and the recovery in all water samples ranged from 78% to 94%.

### 2.2. Derivation of Carbamazepine WQC 

#### 2.2.1. Toxicity Data Collection and Selection

The chronic toxicity data of carbamazepine were selected from the USEPA ECOTOX database (https://cfpub.epa.gov/ecotox/, accessed on 18 May 2022), Web of Science database, and China Knowledge Resource Integrated Database (https://www.cnki.net, accessed on 26 May 2022). No observed effect concentration (NOEC) was the first choice as endpoint; meanwhile, the lowest observed effect concentration (LOEC) and maximum acceptable toxicant concentration (MATC) were also applied when NOEC was not sufficient. The screening criterion for chronic toxicity data referred to previous literature [16].

#### 2.2.2. Derivation WQC of Carbamazepine

WQC is derived by the SSD method, which is widely used throughout the world. Firstly, toxicity values (X axis) and their cumulative frequencies (Y axis) are employed to construct SSD curves. One sample Kolmogorov–Smirnov test is applied to test whether log-transformed field exposure concentrations and toxicity data conformed to normal distribution using SPSS 24.0. The HC_5_ refers to 5% hazard concentration, at which 95% aquatic organisms could be protected, which can be acquired at the tail of the SSD curve. Then, WQC is calculated as HC_5_ divided by an assessment factor of 2 [19].

### 2.3. Ecological Risk Assessment

Three-tiered methods were employed to evaluate the carbamazepine ecological risk in this study.

Tiered 1 was a qualitative risk assessment method called Hazard Quotient (HQ), which was the ratio of environmental exposure concentrations and WQC [30]. The assumption of this method is that a potential negative effect on the aquatic system may occur if the exposure level of a pollutant is greater than its WQC. There are four risk categories which are introduced below.

HQ > 10, the risk is high;

1.0 ≤ HQ < 10, the risk is moderate;

0.1 ≤ HQ < 1.0, the risk is low;

HQ < 0.1, the risk is negligible.

In tiered 2, a quantitative probabilistic risk method named margin of safety (MOS_10_) was applied to describe the risk level [31]. MOS_10_ was calculated using exposure and toxicity data distributions by the following equation:

MOS_10_ = SSD_10_/ECD_90_ (1)

where SSD_10_ is the 10th percentile concentration for the toxicity data distribution, ECD_90_ is the 90th percentile concentration for the environmental exposure distribution. Generally, an MOS_10_ greater than one demonstrates that toxicity data distribution and exposure concentration distribution have a low interval overlap degree, and little environmental risk will be posed to aquatic species, whereas MOS_10_ less than one will lead to a high risk to aquatic organisms.

In tiered 3, a joint probability curve (JPC) method developed based on tiered 2 was used. Exceedance probability function is achieved by transforming the exposure concentration distribution and combining it with the SSD to generate a JPC. JPC describes the relationship between the probability that aquatic species would be affected (X axis) and the exceeded frequency of exposure concentrations (Y axis). The closer JPC is to the X axis, the lower the probability of an adverse effect [32].

## 3. Results and Discussion

### 3.1. Spatial Distribution of Carbamazepine in Nansi Lake Basin

**Figure 2:**
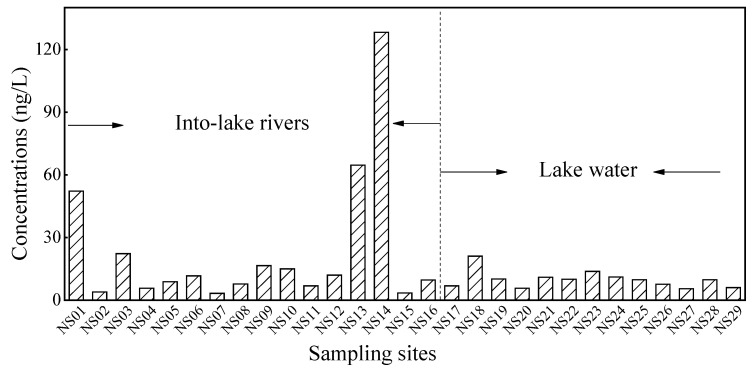
Spatial distribution of carbamazepine in the surface water of Nansi Lake basin.

The concentrations of carbamazepine are illustrated in Figure 2. In all 29 surface water samples, the detection rate of carbamazepine was 100%, which indicated that carbamazepine was ubiquitous in the Nansi Lake basin. The concentrations of carbamazepine from different tributaries ranged from 3.3 to 128.2 ng/L, with an average of 23.3 ng/L; the levels of carbamazepine from lakes varied from 5.5 to 21.1 ng/L, with the mean concentration of 9.9 ng/L. Aqueous residues of carbamazepine were higher than Yellow River (3.57–4.82 ng/L) [33], Taihu Lake (5.16 ng/L) [34], and Dongting Lake (0.06 ng/L) [35]; were in the same level to Baiyangdian Lake (60.3 ng/L) [36]; were lower than that reported in Yangtze River (up to 1090 ng/L) [37] and Hai River (456 ng/L) [38]. Overall, the concentrations of carbamazepine in this study were at a medium level in China (for the detailed exposure data, please see Table S2).

From the perspective of distribution, carbamazepine showed the characteristics of non-homogeneity. In general, the concentration of carbamazepine in the rivers was higher than that in the lakes. The Web of Rxlist (https://www.rxlist.com, accessed on 21th June 2022) showed that 72% of carbamazepine ingested by living organisms was degraded, and the remaining 28% was directly excreted through feces. By field observation, sampling points such as NS01, NS03, NS13, and NS14 were densely populated. Subsequently, carbamazepine was used more frequently. Carbamazepine is a strongly water-soluble compound (112 mg/L) [39]. These may account for the higher concentrations of carbamazepine in several tributaries. Then, the concentration of carbamazepine was reduced after being diluted by water from the lake. Given that carbamazepine is an emerging pollutant, higher concentrations of carbamazepine in the environment will pose a potential risk to aquatic life.

### 3.2. Derivation of WQC for Carbamazepine

**Figure 3:**
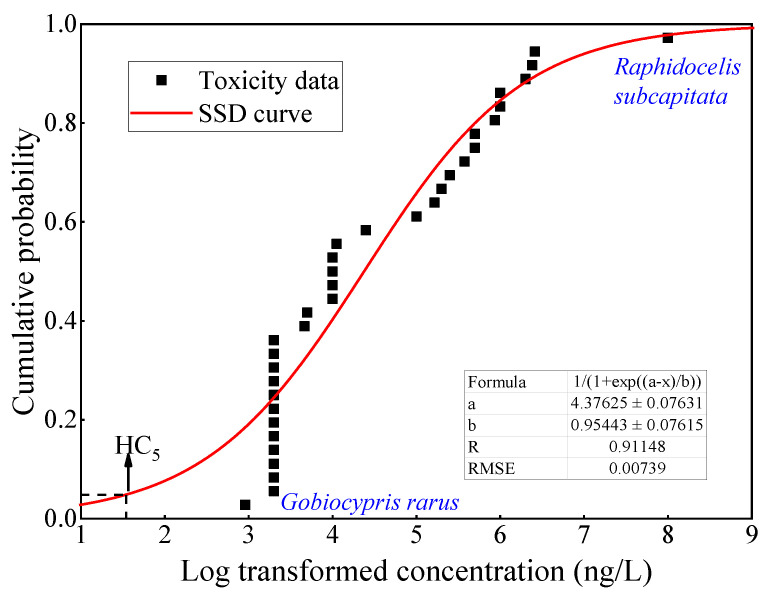
Species sensitivity distributions (SSD) for carbamazepine.

The data of the chronic toxicity of carbamazepine to aquatic organisms are listed in Table S3. The toxicity endpoints were NOEC and LOEC, with the effect indexes of population, growth, behavior, development, etc. Finally, the toxicity data of 35 species from eight taxonomic groups (e.g., amphibians, crustaceans, fishes, algae, higher plants, molluscs, insects, and invertebrates) were screened. The toxicity values of carbamazepine varied from 0.91 to 100,000 μg/L, with the mean concentration of 3201 μg/L, and the most and least sensitive specie was *Gobiocypris rarus* and *Raphidocelis subcapitata*, respectively [40,41]. In this study, the most used extrapolation model based on Log-logistic was used to construct the SSD curves of carbamazepine [42,43,44] (Figure 3). The HC_5_ was calculated to be 36.8 ng/L. In consideration of the uncertainty of deriving WQC, HC_5_ was divided by a factor of 2 as the final carbamazepine WQC, which was 18.4 ng/L.

### 3.3. Ecological Risk Assessment of Carbamazepine

#### 3.3.1. Tiered 1 Assessment

**Figure 4:**
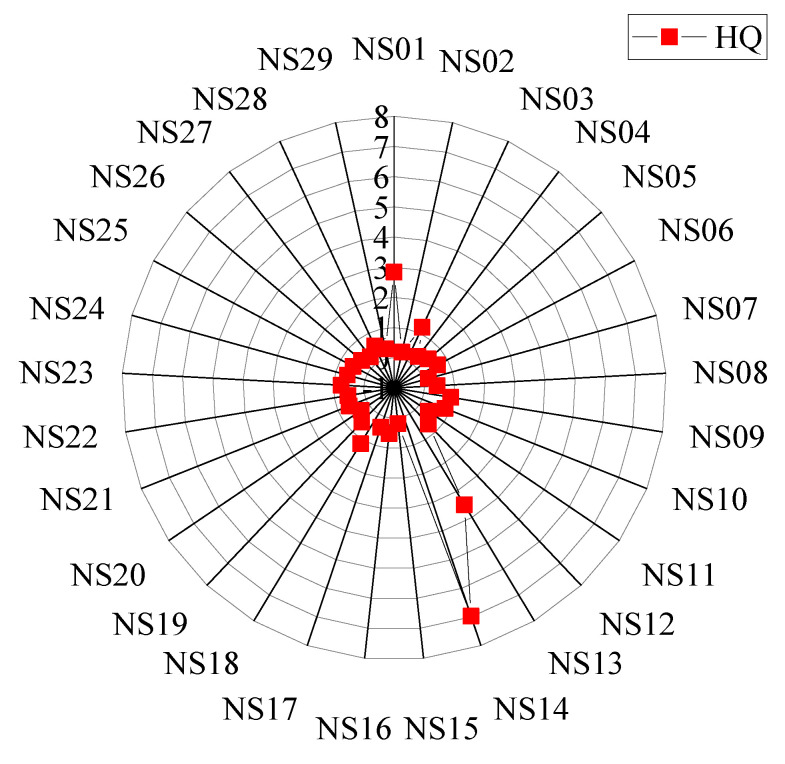
The HQs’ distribution of carbamazepine in Nansi Lake basin.

The HQ method, in which the carbamazepine concentration at each sampling site is divided by the derived WQC, is used to describe the risk level qualitatively. Among the 29 samples, there were 5 with HQs between 1.0 and 10, which were NS01, NS03, NS13, NS14, and NS18, respectively; the other HQs were between 0.1 and 1.0 (Figure 4). The highest risk region was located in NS14, with an HQ of 7.0. In general, the ecological risks of carbamazepine in rivers were higher than those in lakes. The moderate ecological risk areas were mainly concentrated in the eastern and northern parts of the Nansi Lake basin. Considering that the HQ method cannot accurately quantify the degree and probability of ecological risk, tiered 2 and 3 (probabilistic method) are generated subsequently.

#### 3.3.2. Tiered 2 Assessment

**Figure 5:**
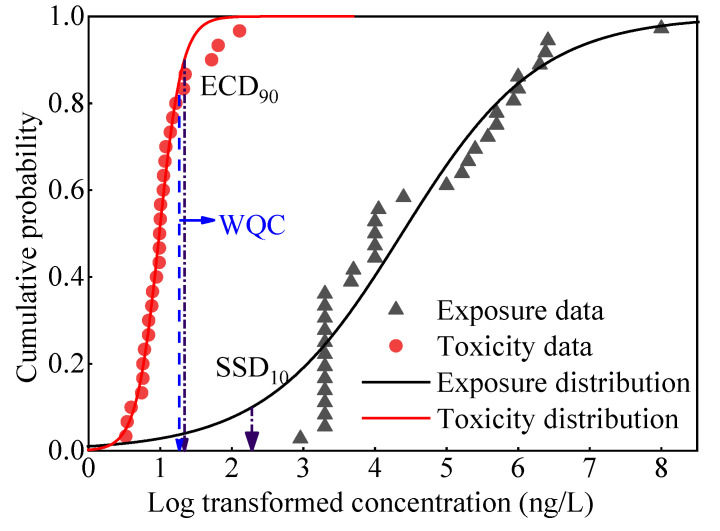
Distribution of exposure concentration and toxicity data of carbamazepine.

As mentioned above, M0S_10_ is the ratio of the 10th concentration of toxicity effect distribution to the 90th concentration of environmental exposure distribution. In this research, M0S_10_ was calculated to be 14.2 (Figure 5). The result showed that carbamazepine did not pose a threat to aquatic species in the Nansi Lake basin. On the other hand, although MOS_10_ was greater than 1, there were still some regions with higher carbamazepine exposure concentration than WQC, indicating that potential ecological risk for aquatic organisms in some waters of the Nansi Lake basin existed.

#### 3.3.3. Tiered 3 Assessment

**Figure 6:**
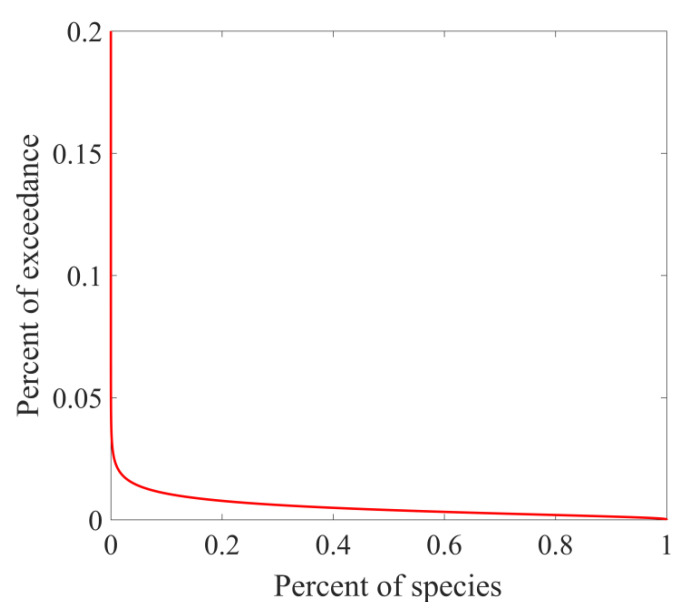
Joint probability curve for ecological risk of carbamazepine in the Nansi Lake basin.

The JPC method is a curve fitted by the concentration of the toxic effect and the concentration of environmental exposure. Compared with HQ and MOS_10_, JPC is a more robust risk assessment method, and can further quantify the ecological environment risk. The Y axis represented the proportion of contaminated surface water bodies, and the X axis represented the proportion of aquatic species being affected (Figure 6). In this study, probabilities of exceeding NOEC or LOEC for 1–5% of the species ranged from 1.4% to 1.0% for carbamazepine in the Nansi Lake basin.

In this study, HQ, MOS_10_, and JPC methods were used to evaluate the risk of carbamazepine to aquatic organisms in the Nansi Lake basin of China. The results all showed that the environmental risk of carbamazepine to the hydrophytic ecosystem was at a low level. The HQ method is a qualitative risk assessment method which is simple to operate and can easily identify the chemical substances that may cause risk to aquatic species. Considering that HQ is a relatively conservative method and the evaluation result is only a single-value estimation which cannot be explained in terms of probability, it is often used as the primary risk assessment. Probabilistic risk assessment methods including MOS_10_ and JPC are developed on the basis of qualitative risk assessment methods, which make full use of all pollutant toxicity data and environmental monitoring data and take into account the effects of other factors on aquatic biosafety, such as the concentration distribution of pollutants, the total amount of pollutants, the type of aquatic organisms, as well as species’ susceptibility to contaminants. For the convenience of environmental management, experts and scholars have suggested the use of qualitative and quantitative risk-assessment methods to assess the ecological risk of pollutants [44].

### 3.4. Uncertainty Analysis

Uncertainty was inevitable in the process of performing ecological risk assessment, which mainly originated from the following factors: carbamazepine environmental exposure, toxicity effect, and the risk characterization method. The uncertainty of environmental exposure included deviation during sample collection and analysis and a lack of information on spatial and temporal variation in carbamazepine field concentrations. Then, the difference between SSD fitting models and limited chronic toxicity data were also vital yet uncertain sources of carbamazepine’s toxicity effect. Furthermore, the NOEC or LOEC used in this study was obtained based on a controlled laboratory experiment, which was somewhat different from that acquired by field experiment. Finally, there were various ecological risk assessment methods, and different researchers had different understandings and applications, which would lead to different risk results in the same situation. Considering these, a tiered method was used to reduce the uncertainty of risk assessment to a large extent.

## 4. Conclusions

Twenty-nine samples from the largest shallow freshwater lake in China were analyzed to identify carbamazepine’s residual and ecological risk to aquatic species. The concentrations of carbamazepine from samples ranged from 3.3 to 128.2 ng/L. The major cause of carbamazepine in the environment might be due to anthropogenic activities. Meanwhile, toxicity data of thirty-five aquatic species were screened to construct SSD curves to determine the WQC of carbamazepine, which was 18.4 ng/L. Moreover, a tiered approach was used to assess carbamazepine’s ecological risk. The HQ method showed that the highest risk region was located in NS14, with an HQ of 7.0. In general, the ecological risks of carbamazepine in rivers were higher than those in lakes. M0S_10_ showed that carbamazepine did not pose a threat to aquatic species in the target area. The JPC method indicated that ecological risks might exist in 1.4% of surface water, while a 5% threshold was set up to protect aquatic species. In this work, both qualitative and quantitative methods indicated that carbamazepine posed a low ecological risk to aquatic systems in the Nansi Lake basin. This study could provide some useful information for decisionmakers to control carbamazepine pollution.

## Data Availability

The data of this study are available from the corresponding author upon reasonable request.

## Supplementary Materials

The following supporting information can be downloaded at: https://www.mdpi.com/article/10.3390/ijerph191710875/s1, Table S1: Instrument parameter for HPLC-MS/MS; Table S2: Carbamazepine occurrence in surface water of China; Table S3: Chronic toxicity of carbamazepine to freshwater animals. References [45,46,47,48,49,50,51,52,53,54,55,56,57,58,59,60,61,62,63,64,65,66,67,68,69,70,71,72] are cited in the supplementary materials.

## References

[1] Kan X., Feng S., Mei X., Sui Q., Zhao W., Lyu S., Sun S., Zhang Z., Yu G. (2022). Quantitatively identifying the emission sources of pharmaceutically active compounds (PhACs) in the surface water: Method development, verification and application in Huangpu River, China. Sci. Total Environ..

[2] Liu N., Jin X., Feng C., Wang Z., Wu F., Johnson A.C., Xiao H., Hollert H., Giesy J.P. (2020). Ecological risk assessment of fifty pharmaceuticals and personal care products (PPCPs) in Chinese surface waters: A proposed multiple-level system. Environ. Int..

[3] Almeida Â., Soares A.M., Esteves V.I., Freitas R. (2021). Occurrence of the antiepileptic carbamazepine in water and bivalves from marine environments: A review. Environ. Toxicol. Pharm..

[4] Chafi S., Azzouz A., Ballesteros E. (2022). Occurrence and distribution of endocrine disrupting chemicals and pharmaceuticals in the river Bouregreg (Rabat, Morocco). Chemosphere.

[5] Waleng N.J., Nomngongo P.N. (2022). Occurrence of pharmaceuticals in the environmental waters: African and Asian perspectives. Environ. Chem. Ecotoxicol..

[6] Dai C., Li S., Duan Y., Leong K.H., Tu Y., Zhou L. (2021). Human health risk assessment of selected pharmaceuticals in the five major river basins, China. Sci. Total Environ..

[7] Fraz S., Lee A.H., Wilson J.Y. (2018). Gemfibrozil and carbamazepine decrease steroid production in zebrafish testes (*Danio rerio*). Aquat. Toxicol..

[8] Lamichhane K., Garcia S.N., Huggett D.B., DeAngelis D.L., La Point T.W. (2013). Chronic effects of carbamazepine on life-history strategies of *Ceriodaphnia dubia* in three successive generations. Arch. Environ. Contam. Toxicol..

[9] Jarvis A.L., Bernot M.J., Bernot R.J. (2014). The effects of the psychiatric drug carbamazepine on freshwater invertebrate communities and ecosystem dynamics. Sci. Total Environ..

[10] Tousova Z., Oswald P., Slobodnik J., Blaha L., Muz M., Hu M., Brack W., Krauss M., Di Paolo C., Tarcai Z. (2017). European demonstration program on the effect-based and chemical identification and monitoring of organic pollutants in European surface waters. Sci. Total Environ..

[11] Feng C., Li H., Yan Z., Wang Y., Wang C., Fu Z., Liao W., Giesy J.P., Bai Y. (2019). Technical study on national mandatory guideline for deriving water quality criteria for the protection of freshwater aquatic organisms in China. J. Environ. Manag..

[12] ANZECC, ARMCANZ (2000). Australian and New Zealand Guidelines for Fresh and Marine Water Quality.

[13] CCME (2007). A Protocol for the Derivation of Water Quality Guidelines for the Protection of Aquatic Life.

[14] ECR (2003). Technical Guidance Document on Risk Assessment in Support of Commission Directive 93/67/EEC on Risk Assessment for New Notified Substances, Commission Regulation (EC) No 1488/94 on Risk Assessment for Existing Substances, and Directive 98/8/EC of the European Parliament and of the Council Concerning the Placing of Biocidal Products on the Market. Part I–IV.

[15] RIVM (2007). Guidance for the Derivation of Environmental Risk Limits within the Framework of ’International and National Environmental Quality Standards for Substances in the Netherlands’ (INS).

[16] USEPA (1985). Guidelines for Deriving Numerical National Water Quality Criteria for the Protection of Aquatic Organisms and Their Uses.

[17] USEPA (2018). National Recommended Water Quality Criteria.

[18] Zheng L., Zhang J., Yan Z. (2016). Development of seawater aquatic life criteria for ammonia in China. Acta Oceanol. Sin..

[19] Zheng L., Liu Z., Yan Z., Yi X., Zhang J., Zhang Y., Zheng X., Zhu Y. (2017). Deriving water quality criteria for trivalent and pentavalent arsenic. Sci. Total Environ..

[20] Yan Z.G., Liu Z.T., Wang H., Liang F., Li J., Liu H., Cheng S., Liang L. (2012). Development of aquatic life criteria for nitrobenzene in China. Environ. Pollut..

[21] Gao X., Li J., Wang X., Zhou J., Fan B., Li W., Liu Z. (2019). Exposure and ecological risk of phthalate esters in the Taihu Lake basin, China. Ecotoxicol. Environ. Saf..

[22] Wu F., Mu Y., Chang H., Zhao X., Giesy J.P., Wu K.B. (2013). Predicting water quality criteria for protecting aquatic life from physicochemical properties of metals or metalloids. Environ. Sci. Technol..

[23] MEE (2017). Technical Guideline for Deriving Water Quality Criteria for the Protection of Freshwater Aquatic Organisms (HJ 831-2017).

[24] MEE (2022). Technical Guideline for Deriving Water Quality Criteria for Freshwater Aquatic Organisms (HJ 831-2022).

[25] Wang L.F., Yang L.Y., Kong L.H., Li S., Zhu J.R., Wang Y.Q. (2014). Spatial distribution, source identification and pollution assessment of metal content in the surface sediments of Nansi Lake, China. J. Geochem. Explor..

[26] Wang W., Liu X., Wang Y., Guo X., Lu S. (2016). Analysis of point source pollution and water environmental quality variation trends in the Nansi Lake basin from 2002 to 2012. Environ. Sci. Pollut. Res..

[27] Li L., Zhang Y., Wang J., Lu S., Cao Y., Tang C., Yan Z., Zheng L. (2020). History traces of HCHs and DDTs by groundwater dating and their behaviours and ecological risk in northeast China. Chemosphere.

[28] Liu J., Dan X., Lu G., Shen J., Wu D., Yan Z. (2018). Investigation of pharmaceutically active compounds in an urban receiving water: Occurrence, fate and environmental risk assessment. Ecotoxicol. Environ. Saf..

[29] Zhang P., Zhou H., Li K., Zhao X., Liu Q., Li D., Zhao G., Wang L. (2018). Occurrence of pharmaceuticals and personal care products, and their associated environmental risks in Guanting Reservoir and its upstream rivers in north China. RSC Adv..

[30] Yan Z., Wang W., Zhou J., Yi X., Zhang J., Wang X., Liu Z. (2015). Screening of high phytotoxicity priority pollutants and their ecological risk assessment in China’s surface waters. Chemosphere.

[31] Zheng L., Liu Z., Yan Z., Zhang Y., Yi X., Zhang J., Xin Z., Zhou J., Yan Z. (2017). pH-dependent ecological risk assessment of pentachlorophenol in Taihu Lake and Liaohe River. Ecotoxicol. Environ. Saf..

[32] Zolezzi M., Cattaneo C., Tarazona J.V. (2005). Probabilistic ecological risk assessment of 1,2,4-trichlorobenzene at a former industrial contaminated site. Environ. Sci. Technol..

[33] Feng J., Liu Q., Ru X., Xi N., Sun J. (2020). Occurrence and distribution of priority pharmaceuticals in the Yellow River and the Huai River in Henan, China. Environ. Sci. Pollut. Res..

[34] An W., Duan L., Zhang Y., Zhou Y., Wang B., Yu G. (2022). Pollution characterization of pharmaceutically active compounds (PhACs) in the northwest of Tai Lake Basin, China: Occurrence, temporal changes, riverine flux and risk assessment. J. Hazard. Mater..

[35] Wang Y., Liu Y., Lu S., Liu X., Meng Y., Zhang G., Zhang Y., Wang W., Guo X. (2019). Occurrence and ecological risk of pharmaceutical and personal care products in surface water of the Dongting Lake, China-during rainstorm period. Environ. Sci. Pollut. Res..

[36] Yang L., Wang T., Zhou Y., Shi B., Bi R., Meng J. (2021). Contamination, source and potential risks of pharmaceuticals and personal products (PPCPs) in Baiyangdian Basin, an intensive human intervention area, China. Sci. Total Environ..

[37] Zhou X., Dai C., Zhang Y., Surampalli R., Zhang T. (2011). A preliminary study on the occurrence and behavior of carbamazepine (CBZ) in aquatic environment of Yangtze River Delta, China. Environ. Monit. Assess..

[38] Zhou H., Wu C., Huang X., Gao M., Wen X., Tsuno H., Tanaka H. (2010). Occurrence of selected pharmaceuticals and caffeine in sewage treatment plants and receiving rivers in Beijing, China. Water Environ. Res..

[39] Zhang L., Du S., Zhang X., Lyu G., Dong D., Hua X., Zhang W., Guo Z. (2020). Occurrence, distribution, and ecological risk of pharmaceuticals in a seasonally ice-sealed river: From ice formation to melting. J. Hazard. Mater..

[40] Ferrari B.T., Paxéus N., Giudice R.L., Pollio A., Garric J. (2003). Ecotoxicological impact of pharmaceuticals found in treated wastewaters: Study of carbamazepine, clofibric acid, and diclofenac. Ecotoxicol. Environ. Saf..

[41] Yan S., Chen R., Wang M., Zha J. (2021). Carbamazepine at environmentally relevant concentrations caused DNA damage and apoptosis in the liver of Chinese rare minnows (*Gobiocypris rarus*) by the Ras/Raf/ERK/p53 signaling pathway. Environ. Pollut..

[42] Awkerman J.A., Raimondo S., Barron M.G. (2008). Development of Species Sensitivity Distributions for Wildlife using Interspecies Toxicity Correlation Models. Environ. Sci. Technol..

[43] Dyer S.D., Versteeg D.J., Belanger S.E., Chaney J.G., Raimondo S., Barron M.G. (2008). Comparison of species sensitivity distributions derived from interspecies correlation models to distributions used to derive water quality criteria. Environ. Sci. Technol..

[44] Zheng X., Yan Z., Liu P., Li H., Zhou J., Wang Y., Fan J., Liu Z. (2019). Derivation of aquatic life criteria for four phthalate esters and their ecological risk assessment in Liao River. Chemosphere.

[45] Wu C., Huang X., Witter J.D., Spongberg A.L., Wang K., Wang D., Liu J. (2014). Occurrence of pharmaceuticals and personal care products and associated environmental risks in the central and lower Yangtze river, China. Ecotoxicol. Environ. Saf..

[46] Liu J., Lu G., Xie Z., Zhang Z., Li S., Yan Z. (2015). Occurrence, bioaccumulation and risk assessment of lipophilic pharmaceutically active compounds in the downstream rivers of sewage treatment plants. Sci. Total Environ..

[47] Zhou H., Ying T., Wang X., Liu J. (2016). Occurrence and preliminarily environmental risk assessment of selected pharmaceuticals in the urban rivers, China. Sci. Rep..

[48] Sun J., Luo Q., Wang D., Wang Z. (2015). Occurrences of pharmaceuticals in drinking water sources of major river watersheds, China. Ecotoxicol. Environ. Saf..

[49] Yang J.-F., Ying G.-G., Zhao J.-L., Tao R., Su H.-C., Liu Y.-S. (2011). Spatial and seasonal distribution of selected antibiotics in surface waters of the Pearl Rivers, China. J. Environ. Sci. Health B.

[50] Yu Y., Huang Q., Wang Z., Zhang K., Tang C., Cui J., Feng J., Peng X. (2011). Occurrence and behavior of pharmaceuticals, steroid hormones, and endocrine-disrupting personal care products in wastewater and the recipient river water of the Pearl River Delta, South China. J. Environ. Monit..

[51] Zhao J.L., Ying G.G., Liu Y.S., Chen F., Yang J.F., Wang L., Yang X.B., Stauber J.L., Warne M.S.J. (2010). Occurrence and a screening-level risk assessment of human pharmaceuticals in the Pearl River system, South China. Environ. Toxicol. Chem..

[52] Ding Z., He D., Wan D., Wu G., Zhang S. (2015). Determination of thirteen pharmaceutical and personal care products in surface water by liquid chromatography-tandem mass spectrometry. Chin. J. Environ. Eng..

[53] Dai G., Wang B., Huang J., Dong R., Deng S., Yu G. (2015). Occurrence and source apportionment of pharmaceuticals and personal care products in the Beiyun River of Beijing, China. Chemosphere.

[54] Ma R., Wang B., Yin L., Zhang Y., Deng S., Huang J., Wang Y., Yu G. (2017). Characterization of pharmaceutically active compounds in Beijing, China: Occurrence pattern, spatiotemporal distribution and its environmental implication. J. Hazard. Mater..

[55] Dai G., Wang B., Fu C., Dong R., Huang J., Deng S., Wang Y., Yu G. (2016). Pharmaceuticals and personal care products (PPCPs) in urban and suburban rivers of Beijing, China: Occurrence, source apportionment and potential ecological risk. Environ. Sci-Proc. Imp..

[56] Zhu S., Chen H., Li J. (2013). Sources, distribution and potential risks of pharmaceuticals and personal care products in Qingshan Lake basin, Eastern China. Ecotoxicol. Environ. Saf..

[57] Lawrence J.R., Swerhone G.D., Wassenaar L.I., Neu T.R. (2005). Effects of selected pharmaceuticals on riverine biofilm communities. Can. J. Microbiol..

[58] Jarvis A.L., Bernot M.J., Bernot R.J. (2014). The effects of the pharmaceutical carbamazepine on life history characteristics of flat-headed mayflies (Heptageniidae) and aquatic resource interactions. Ecotoxicology.

[59] Zhang W., Zhang M., Lin K., Sun W., Xiong B., Guo M., Cui X., Fu R. (2012). Eco-toxicological effect of Carbamazepine on *Scenedesmus obliquus* and *Chlorella pyrenoidosa*. Environ. Toxicol. Pharm..

[60] Haase S.M., Panas P., Rath T., Huchzermeyer B. (2015). Effects of carbamazepine on two microalgae species differing in stress resistance. Water Air Soil Pollut..

[61] Melvin S.D., Cameron M.C., Lanctôt C.M. (2014). Individual and mixture toxicity of pharmaceuticals naproxen, carbamazepine, and sulfamethoxazole to Australian striped marsh frog tadpoles (*Limnodynastes peronii*). J. Toxicol. Environ. Health A.

[62] Dussault È.B., Balakrishnan V.K., Sverko E., Solomon K.R., Sibley P.K. (2008). Toxicity of human pharmaceuticals and personal care products to benthic invertebrates. Environ. Toxicol. Chem..

[63] Lürling M., Sargant E., Roessink I. (2006). Life—History consequences for *Daphnia pulex* exposed to pharmaceutical carbamazepine. Environ. Toxicol..

[64] Jubeaux G., Simon R., Salvador A., Quéau H., Chaumot A., Geffard O. (2012). Vitellogenin-like proteins in the freshwater amphipod *Gammarus fossarum* (Koch, 1835): Functional characterization throughout reproductive process, potential for use as an indicator of oocyte quality and endocrine disruption biomarker in males. Aquat. Toxicol..

[65] Overturf M., Overturf C., Baxter D., Hala D., Constantine L., Venables B., Huggett D. (2012). Early life-stage toxicity of eight pharmaceuticals to the fathead minnow, *Pimephales promelas*. Arch. Environ. Contam. Toxicol..

[66] Triebskorn R., Casper H., Scheil V., Schwaiger J. (2007). Ultrastructural effects of pharmaceuticals (carbamazepine, clofibric acid, metoprolol, diclofenac) in rainbow trout (*Oncorhynchus mykiss*) and common carp (*Cyprinus carpio*). Anal. Bioanal. Chem..

[67] Li Z.H., Zlabek V., Velisek J., Grabic R., Machova J., Randak T. (2010). Physiological condition status and muscle-based biomarkers in rainbow trout (*Oncorhynchus mykiss*), after long-term exposure to carbamazepine. J. Appl. Toxicol..

[68] Dordio A., Belo M., Teixeira D.M., Carvalho A.P., Dias C., Picó Y., Pinto A.P. (2011). Evaluation of carbamazepine uptake and metabolization by *Typha* spp., a plant with potential use in phytotreatment. Bioresour. Technol..

[69] Brain R.A., Johnson D.J., Richards S.M., Hanson M.L., Sanderson H., Lam M.W., Young C., Mabury S.A., Sibley P.K., Solomon K.R. (2004). Microcosm evaluation of the effects of an eight pharmaceutical mixture to the aquatic macrophytes *Lemna gibba* and *Myriophyllum sibiricum*. Aquat. Toxicol..

[70] Oetken M., Nentwig G., Löffler D., Ternes T., Oehlmann J. (2005). Effects of pharmaceuticals on aquatic invertebrates. Part I. The antiepileptic drug carbamazepine. Arch. Environ. Contam. Toxicol..

[71] Nentwig G., Oetken M., Oehlmann J. (2004). Effects of Pharmaceuticals on Aquatic Invertebrates—The Example of Carbamazepine and Clofibric Acid. Pharmaceuticals in the Environment.

[72] Chen H., Zha J., Liang X., Li J., Wang Z. (2014). Effects of the human antiepileptic drug carbamazepine on the behavior, biomarkers, and heat shock proteins in the Asian clam *Corbicula fluminea*. Aquat. Toxicol..

