# Supplementary material for: Derivation of Water Quality Criteria for Carbamazepine and Ecological Risk Assessment in the Nansi Lake Basin
Source: Int J Environ Res Public Health. 2022 Aug 31;19(17):10875. doi: 10.3390/ijerph191710875 (PMC9518526; doi:10.3390/ijerph191710875)
Supplement: Supplementary file 1 [file ijerph-19-10875-s001.zip › ijerph-1834001-supplementary.pdf]

Derivation of water quality criteria for carbamazepine and ecological risk assessment in the Nansi Lake basin

Jiangyue Wu [a], Dianlong Shi [b,], Sai Wang [b], Xi Yang [b], Hui Zhang [b], Ting Zhang [b], Lei Zheng [b]*, Yizhang Zhang [c, d]*

[a] *National Marine Hazard Mitigation Service, Ministry of Natural Resource of the People's Republic of China, Beijing 100194, China*

[b] *State Environmental Protection Key laboratory of Dioxin Pollution, National Research Center of Environmental Analysis and Measurement, Sino-Japan Friendship Center for Environmental Protection, Beijing 100029, China*

[c] *Chinese Research Academy of Environmental Sciences, Beijing 100012, China*

[d] *Research Institute for Environmental Innovation (Tianjin Binhai), Tianjin 300457, China*

* Corresponding authors. Tel: +86 10 84665751, Fax: +86 10 84634275

E-mail addresses: zhenglei@edcmep.org.cn, zhangyz@craes.org.cn (Y. Zhang)

Table S1 Instrument parameter for HPLC-MS/MS

**LC parameter**	**LC setting**	**MS/MS parameter**	**ICP-MS settings**
Column	Waters UPLC BEH C_{18} (50 mm × 2.1 mm, 1.7 μm)	Mode	MRM
Mobile phase A	ultrapure water with 0.01% formic acid	Ion source	ESI
Mobile phase B	Methanol	Capillary voltage	ESI^+ 3.0 kv
Flow rate	0.3 mL/min: 0–2 min, 20% B to 60% B; 2–10 min, 60% to 100% B; remain 4 min.	Parent ion (m/z)	195.1
Injected volume	10 μL	Daughter ion (m/z)	138.2
Column temperature	40℃	Cone voltage	22 v
		Collision energy	17 eV

Table S2 Carbamazepine occurrence in surface water of China

Sampling site	Types	Time	Concentration (ng/L)	Average (ng/L)	Reference
Yangtze River	River	January and March 2013	1.87	88.16	[1]
		July 2008	362		[2]
		October 2013	2.00		[3]
		2014-2015	13.0		[4]
		cold season 2012	0.09		[5]
		warm season 2013	0.16		[5]
		November 2009	238		[6]
Yellow River	River	cold season 2012	0.33	2.97	[5]
		warm season 2013	1.60		[5]
		Normal season 2014	4.82		[7]
		Wet season 2014	3.57		[7]
		Dry season 2014	4.53		[7]
Pearl River	River	May 2008	15.0	10.48	[8]
		/	15.6		[9]
		/	0.83		[10]
Hai River	River	March 2013	56.4	89.73	[11]
		cold season 2012	0.45		[5]
		warm season 2013	0.29		[5]
		July and November of 2015	0.53		[12]
		May 2014	24.7		[13]
		July 2008	456		[14]
Huai River	River	cold season 2012	1.73	2.69	[5]
		warm season 2013	1.03		[5]
		Normal season	3.25		[7]

Sampling site	Types	Time	Concentration (ng/L)	Average (ng/L)	Reference
		2014			
		Wet season 2014	2.92		[7]
		Dry season 2013	4.53		[7]
Songliao River	River	cold season 2012	0.07	0.90	[5]
		warm season 2013	0.09		[5]
		dry season	2.44		[15]
		2018-2019	1.0		[16]
Chao lake	Lake	January and March 2013	2.70	2.70	[1]
Dongting lake	Lake	January and March 2013	2.30	1.18	[1]
		2017	0.06		[17]
Poyang lake	Lake	January and March 2013	2.60	2.60	[1]
Tai lake	Lake	January and March 2013	1.30	3.23	[1]
		2019	5.16		[18]
Qingshan Lake	Lake	dry season	4.20	1.67	[15]
		median water season	0.22		[15]
		wet season	0.59		[15]
Baiyangdian	Lake	May 2019	60.3	36.9	[19]
		November 2019	13.5		[19]

Table S3 Chronic toxicity of Carbamazepine to freshwater animals

Species	Taxonomic Group	Exposure Type	Duration Days (d)	Effect	Endpoint	Concentrations (ng/L)	Reference
Monera	Algae	Flow-through	56	Population	LOEC	10000	[20]
Chaetophora sp.	Algae	Static	9	Population	NOEC	2000	[21]
Raphidocelis subcapitata	Algae	Not reported	4	Population	NOEC	100000000	[22]
Scenedesmus acutus var. acutus	Algae	Not reported	30	Population	NOEC	500000	[23]
Parachlorella kessleri	Algae	Static	3	Population	NOEC	10000	[24]
Chlorella pyrenoidosa	Algae	Not reported	30	Population	NOEC	500000	[23]
Algae sp.	Algae	Flow-through	56	Population	NOEC	10000	[20]
Limnodynastes peronii	Amphibians	Renewal	21	Development	NOEC	10000	[25]
Ceriodaphnia sp.	Crustaceans	Static	31	Population	NOEC	2000	[26]
Calanoida	Crustaceans	Static	31	Population	NOEC	2000	[26]

Species	Taxonomic Group	Exposure Type	Duration Days (d)	Effect	Endpoint	Concentrations (ng/L)	Reference
Cyclopoida	Crustaceans	Static	31	Population	NOEC	2000	[26]
Chydorus sp.	Crustaceans	Static	31	Population	NOEC	2000	[26]
Hyalella azteca	Crustaceans	Renewal	10	Growth	EC10	2400000	[27]
Ceriodaphnia dubia	Crustaceans	Renewal	7	Reproduction	NOEC	25000	[22]
Daphnia pulex	Crustaceans	Renewal	14	Reproduction	NOEC	100000	[28]
Gammarus fossarum	Crustaceans	Renewal	21	Biochemistry	NOEL	1000000	[29]
Gobiocypris rarus	Fish	Flow-through	28	Enzyme(s)	NOEC	910	[30]
Danio rerio	Fish	Renewal	42	Reproduction	LOEC	11200	[31]
Pimephales promelas	Fish	Renewal	28	Growth	NOEC	862000	[32]
Cyprinus carpio	Fish	Flow-through	28	Histology	NOEC	5000	[33]
Oncorhynchus mykiss	Fish	Renewal	42	Growth	NOEC	200000	[34]
Plantae	Plants	Static	31	Population	NOEC	2000	[26]
Typha sp.	Plants	Not reported	21	Growth	NOEC	2000000	[35]

Species	Taxonomic Group	Exposure Type	Duration Days (d)	Effect	Endpoint	Concentrations (ng/L)	Reference
Lemna gibba	Plants	Renewal	7	Injury	NOEC	1000000	[36]
Chironomus tentans	Insects	Renewal	10	Growth	EC10	2600000	[27]
Chironomus riparius	Insects	Static	28	Development	NOEC	164000	[37]
Invertebrates	Invertebrates	Static	31	Population	NOEC	2000	[26]
Brachionus calyciflorus	Invertebrates	Not reported	2	Mortality	NOEC	377000	[22]
Gastropoda	Molluscs	Static	31	Population	NOEC	2000	[26]
Planorbella trivolvis	Molluscs	Static	31	Population	NOEC	2000	[26]
Physella acuta	Molluscs	Static	31	Population	NOEC	2000	[26]
Elimia livescens	Molluscs	Static	31	Population	NOEC	2000	[26]
Lymnaea stagnalis	Molluscs	Static	31	Population	NOEC	2000	[26]
Potamopyrgus antipodarum	Molluscs	Renewal	28	Reproduction	NOEC	250000	[38]
Corbicula manilensis	Molluscs	Renewal	30	Behavior	NOEC	4620	[39]

Reference

1. Wu, C., Huang, X., Witter, J.D., Spongberg, A.L., Wang, K., Wang, D., Liu, J. Occurrence of pharmaceuticals and personal care products and associated environmental risks in the central and lower Yangtze river, China. *Ecotox. Environ. Safe.* **2014**, 106, 19-26. https://doi.org/10.1016/j.ecoenv.2014.04.029.
2. Zhou, X., Dai, C., Zhang, Y., Surampalli, R., Zhang, T. A preliminary study on the occurrence and behavior of carbamazepine (CBZ) in aquatic environment of Yangtze River Delta, China. *Environ. Monit. Assess.* **2011**, 173, 45-53. https://doi.org/10.1007/s10661-010-1369-8.
3. Liu, J., Lu, G., Xie, Z., Zhang, Z., Li, S., Yan, Z. Occurrence, bioaccumulation and risk assessment of lipophilic pharmaceutically active compounds in the downstream rivers of sewage treatment plants. *Sci. Total. Environ.* **2015**, 511, 54-62. https://doi.org/10.1016/j.scitotenv.2014.12.033.
4. Zhou, H., Ying, T., Wang, X., Liu, J. Occurrence and preliminarily environmental risk assessment of selected pharmaceuticals in the urban rivers, China. *Sci. Rep-UK.* **2016**, 6, 1-10. https://doi.org/10.1038/srep34928.
5. Sun, J., Luo, Q., Wang, D., Wang, Z. Occurrences of pharmaceuticals in drinking water sources of major river watersheds, China. *Ecotox. Environ. Safe.* **2015**, 117, 132-140. https://doi.org/10.1016/j.ecoenv.2015.03.032.
6. Yang, J.-F., Ying, G.-G., Zhao, J.-L., Tao, R., Su, H.-C., Liu, Y.-S. Spatial and seasonal distribution of selected antibiotics in surface waters of the Pearl Rivers, China. *J. Environ. Sci. Heal. B* **2011**, 46, 272-280. https://doi.org/10.1080/03601234.2011.540540.
7. Feng, J., Liu, Q., Ru, X., Xi, N., Sun, J. Occurrence and distribution of priority pharmaceuticals in the Yellow River and the Huai River in Henan, China. *Environ. Sci. Pollut. Res.* **2020**, 27, 16816-16826. https://doi.org/10.1007/s11356-020-08131-6.
8. Yu, Y., Huang, Q., Wang, Z., Zhang, K., Tang, C., Cui, J., Feng, J., Peng, X. Occurrence and behavior of pharmaceuticals, steroid hormones, and endocrine-disrupting personal care products in wastewater and the recipient river water of the Pearl River Delta, South China. *J. Environ. Monitor.* **2011**, 13, 871-878. https://doi.org/10.1039/C0EM00602E.
9. Zhao, J.L., Ying, G.G., Liu, Y.S., Chen, F., Yang, J.F., Wang, L., Yang, X.B., Stauber, J.L., Warne, M.S.J. Occurrence and a screening - level risk assessment of human pharmaceuticals in the Pearl River system, South China. *Environ. Toxicol. Chem.* **2010**, 29, 1377-1384. https://doi.org/10.1002/etc.161.
10. Ding, Z., He, D., Wan, D., Wu, G., Zhang, S. Determination of thirteen pharmaceutical and personal care products in surface water by liquid chromatography-tandem mass spectrometry. *Chin. J. Environ. Eng.* **2015**, 9, 2291-2296. (in Chinese).
11. Dai, G., Wang, B., Huang, J., Dong, R., Deng, S., Yu, G. Occurrence and source apportionment of pharmaceuticals and personal care products in the Beiyun River of Beijing, China. *Chemosphere* **2015**, 119, 1033-1039. https://doi.org/10.1016/j.chemosphere.2014.08.056.
12. Ma, R., Wang, B., Yin, L., Zhang, Y., Deng, S., Huang, J., Wang, Y., Yu, G. Characterization of pharmaceutically active compounds in Beijing, China: occurrence pattern, spatiotemporal distribution and its environmental implication. *J. Hazard. Mater.* **2017**, 323, 147-155.

https://doi.org/10.1016/j.jhazmat.2016.05.030.
13. Dai, G., Wang, B., Fu, C., Dong, R., Huang, J., Deng, S., Wang, Y., Yu, G. Pharmaceuticals and personal care products (PPCPs) in urban and suburban rivers of Beijing, China: occurrence, source apportionment and potential ecological risk. *Environ. Sci-Proc. Imp.* **2016**, 18, 445-455. https://doi.org/10.1039/C6EM00018E.
14. Zhou, H., Wu, C., Huang, X., Gao, M., Wen, X., Tsuno, H., Tanaka, H. Occurrence of selected pharmaceuticals and caffeine in sewage treatment plants and receiving rivers in Beijing, China. *Water Environ. Res.* **2010**, 82, 2239-2248. https://doi.org/10.2175/106143010X12681059116653.
15. Zhu, S., Chen, H., Li, J. Sources, distribution and potential risks of pharmaceuticals and personal care products in Qingshan Lake basin, Eastern China. *Ecotox. Environ. Safe.* **2013**, 96, 154-159. https://doi.org/10.1016/j.ecoenv.2013.06.033.
16. Zhang, L., Du, S., Zhang, X., Lyu, G., Dong, D., Hua, X., Zhang, W., Guo, Z. Occurrence, distribution, and ecological risk of pharmaceuticals in a seasonally ice-sealed river: From ice formation to melting. *J. Hazard. Mater.* **2020**, 389, 122083. https://doi.org/10.1016/j.jhazmat.2020.122083.
17. Wang, Y., Liu, Y., Lu, S., Liu, X., Meng, Y., Zhang, G., Zhang, Y., Wang, W., Guo, X. Occurrence and ecological risk of pharmaceutical and personal care products in surface water of the Dongting Lake, China-during rainstorm period. *Environ. Sci. Pollut. Res.* **2019**, 26, 28796-28807. https://doi.org/10.1007/s11356-019-06047-4.
18. An, W., Duan, L., Zhang, Y., Zhou, Y., Wang, B., Yu, G. Pollution characterization of pharmaceutically active compounds (PhACs) in the northwest of Tai Lake Basin, China: Occurrence, temporal changes, riverine flux and risk assessment. *J. Hazard. Mater.* **2022**, 422, 126889. https://doi.org/10.1016/j.jhazmat.2021.126889.
19. Yang, L., Wang, T., Zhou, Y., Shi, B., Bi, R., Meng, J. Contamination, source and potential risks of pharmaceuticals and personal products (PPCPs) in Baiyangdian Basin, an intensive human intervention area, China. *Sci. Total. Environ.* **2021**, 760, 144080. https://doi.org/10.1016/j.scitotenv.2020.144080.
20. Lawrence, J.R., Swerhone, G.D., Wassenaar, L.I., Neu, T.R. Effects of selected pharmaceuticals on riverine biofilm communities. *Can. J. Microbiol.* **2005**, 51, 655-669. https://doi.org/10.1139/w05-047.
21. Jarvis, A.L., Bernot, M.J., Bernot, R.J. The effects of the pharmaceutical carbamazepine on life history characteristics of flat-headed mayflies (Heptageniidae) and aquatic resource interactions. *Ecotoxicology* **2014**, 23, 1701-1712. https://doi.org/10.1007/s10646-014-1309-4.
22. Ferrari, B.t., Paxéus, N., Giudice, R.L., Pollio, A., Garric, J. Ecotoxicological impact of pharmaceuticals found in treated wastewaters: study of carbamazepine, clofibric acid, and diclofenac. *Ecotox. Environ. Safe.* **2003**, 55, 359-370. https://doi.org/10.1016/S0147-6513(02)00082-9.
23. Zhang, W., Zhang, M., Lin, K., Sun, W., Xiong, B., Guo, M., Cui, X., Fu, R. Eco-toxicological effect of Carbamazepine on *Scenedesmus obliquus* and *Chlorella pyrenoidosa*. *Environ. Toxicol. Pharm.* **2012**, 33, 344-352. https://doi.org/10.1016/j.etap.2011.12.024.
24. Haase, S.M., Panas, P., Rath, T., Huchzermeyer, B. Effects of carbamazepine on two

microalgae species differing in stress resistance. *Water Air Soil Poll.* **2015**, 226, 1-12. https://doi.org/10.1007/s11270-015-2562-8.

25. Melvin, S.D., Cameron, M.C., Lanctôt, C.M. Individual and mixture toxicity of pharmaceuticals naproxen, carbamazepine, and sulfamethoxazole to Australian striped marsh frog tadpoles (*Limnodynastes peronii*). *J. Toxicol. Env. Heal. A* **2014**, 77, 337-345. https://doi.org/10.1080/15287394.2013.865107.
26. Jarvis, A.L., Bernot, M.J., Bernot, R.J. The effects of the psychiatric drug carbamazepine on freshwater invertebrate communities and ecosystem dynamics. *Sci. Total. Environ.* **2014**, 496, 461-470. https://doi.org/10.1016/j.scitotenv.2014.07.084.
27. Dussault, È.B., Balakrishnan, V.K., Sverko, E., Solomon, K.R., Sibley, P.K. Toxicity of human pharmaceuticals and personal care products to benthic invertebrates. *Environ. Toxicol. Chem.* **2008**, 27, 425-432. https://doi.org/10.1897/07-354R.1.
28. Lürling, M., Sargant, E., Roessink, I. Life - history consequences for *Daphnia pulex* exposed to pharmaceutical carbamazepine. *Environ. Toxicol.* **2006**, 21, 172-180. https://doi.org/10.1002/tox.20171.
29. Jubeaux, G., Simon, R., Salvador, A., Quéau, H., Chaumot, A., Geffard, O. Vitellogenin-like proteins in the freshwater amphipod *Gammarus fossarum* (Koch, 1835): functional characterization throughout reproductive process, potential for use as an indicator of oocyte quality and endocrine disruption biomarker in males. *Aquat. Toxicol.* **2012**, 112, 72-82. https://doi.org/10.1016/j.aquatox.2012.01.011.
30. Yan, S., Chen, R., Wang, M., Zha, J. Carbamazepine at environmentally relevant concentrations caused DNA damage and apoptosis in the liver of Chinese rare minnows (*Gobiocypris rarus*) by the Ras/Raf/ERK/p53 signaling pathway. *Environ. Pollut.* **2021**, 270, 116245. https://doi.org/10.1016/j.envpol.2020.116245.
31. Fraz, S., Lee, A.H., Wilson, J.Y. Gemfibrozil and carbamazepine decrease steroid production in zebrafish testes (*Danio rerio*). *Aquat. Toxicol.* **2018**, 198, 1-9. https://doi.org/10.1016/j.aquatox.2018.02.006.
32. Overturf, M., Overturf, C., Baxter, D., Hala, D., Constantine, L., Venables, B., Huggett, D. Early life-stage toxicity of eight pharmaceuticals to the fathead minnow, *Pimephales promelas*. *Arch. Environ. Con. Tox.* **2012**, 62, 455-464. https://doi.org/10.1007/s00244-011-9723-6.
33. Triebskorn, R., Casper, H., Scheil, V., Schwaiger, J. Ultrastructural effects of pharmaceuticals (carbamazepine, clofibric acid, metoprolol, diclofenac) in rainbow trout (*Oncorhynchus mykiss*) and common carp (*Cyprinus carpio*). *Anal. Bioanal. Chem.* **2007**, 387, 1405-1416. https://doi.org/10.1007/s00216-006-1033-x.
34. Li, Z.H., Zlabek, V., Velisek, J., Grabic, R., Machova, J., Randak, T. Physiological condition status and muscle - based biomarkers in rainbow trout (*Oncorhynchus mykiss*), after long - term exposure to carbamazepine. *J. Appl. Toxicol.* **2010**, 30, 197-203. https://doi.org/10.1002/jat.1482.
35. Dordio, A., Belo, M., Teixeira, D.M., Carvalho, A.P., Dias, C., Picó, Y., Pinto, A.P. Evaluation of carbamazepine uptake and metabolization by *Typha* spp., a plant with potential use in phytotreatment. *Bioresource Technol.* **2011**, 102, 7827-7834. https://doi.org/10.1016/j.biortech.2011.06.050.
36. Brain, R.A., Johnson, D.J., Richards, S.M., Hanson, M.L., Sanderson, H., Lam, M.W., Young,

C., Mabury, S.A., Sibley, P.K., Solomon, K.R. Microcosm evaluation of the effects of an eight pharmaceutical mixture to the aquatic macrophytes *Lemna gibba* and *Myriophyllum sibiricum*. *Aquat. Toxicol.* **2004**, 70, 23-40. https://doi.org/10.1016/j.aquatox.2004.06.011.

37. Oetken, M., Nentwig, G., Löffler, D., Ternes, T., Oehlmann, J. Effects of pharmaceuticals on aquatic invertebrates. Part I. The antiepileptic drug carbamazepine. *Arch. Environ. Con. Tox.* **2005**, 49, 353-361. https://doi.org/10.1007/s00244-004-0211-0.

38. Nentwig, G., Oetken, M., Oehlmann, J., 2004. Effects of pharmaceuticals on aquatic invertebrates - the example of carbamazepine and clofibric acid, Pharmaceuticals in the Environment. Springer,, pp. 195-208. https://doi.org/10.1007/978-3-662-09259-0_16.

39. Chen, H., Zha, J., Liang, X., Li, J., Wang, Z. Effects of the human antiepileptic drug carbamazepine on the behavior, biomarkers, and heat shock proteins in the Asian clam *Corbicula fluminea*. *Aquat. Toxicol.* **2014**, 155, 1-8. https://doi.org/10.1016/j.aquatox.2014.06.001.
